# Determinants of accuracy in frame-based stereoelectroencephalography: the role of experience, workflow, and selective use of guiding devices

**DOI:** 10.3389/fneur.2026.1796582

**Published:** 2026-03-20

**Authors:** Sabrina V. Kirchleitner, Stefanie Quach, Sebastian Niedermeyer, Pia Nerlinger, Anne Nack, Christian Vollmar, Jan Remi, Hanna Zimmermann, Niklas Thon, Michael Schmutzer-Sondergeld

**Affiliations:** 1 Department of Neurosurgery, LMU University Hospital, LMU Munich, Munich, Germany; 2 Department of Neurosurgery, University Hospital Marburg, Philipps-University Marburg, Marburg, Germany; 3 Department of Neurosurgery, University Hospital OWL, Campus Bielefeld Hospital, Bielefeld, Germany; 4 Department of Neurosurgery, University Hospital Knappschaftskrankenhaus, Ruhr-Universität Bochum, Bochum, Germany; 5 Department of Neurology, LMU University Hospital, LMU Munich, Munich, Germany; 6 Department for Diagnostic and Interventional Neuroradiology, LMU University Hospital, LMU Munich, Munich, Germany

**Keywords:** epilepsy surgery, frame-based stereotaxy, learning curve, slotted guiding cannula, Stereoelectroencephalography (SEEG), workflow optimization

## Abstract

**Background:**

Stereoelectroencephalography (sEEG) is an essential diagnostic procedure for patients with drug-resistant epilepsy. Accurate electrode placement is critical for reliable seizure localization and minimizing complications. The slotted guiding cannula has been proposed as an adjunct to improve implantation accuracy, though its clinical benefit remains uncertain.

**Objective:**

This study evaluates the accuracy, safety, and complication profile of sEEG implantations performed with and without a slotted guiding cannula.

**Methods:**

In this retrospective, single-center cohort study, 59 sEEG procedures were analyzed, encompassing 678 electrodes implanted using the Leksell G-frame system between September 2021 and May 2025. Postoperative CT imaging was fused with preoperative planning data to assess deviations at entry, target, and depth. Accuracy, complication rates, and workflow-related factors such as experience and implantation sequence were evaluated statistically.

**Results:**

Mean deviations were 0.35 ± 1.20 mm at entry, 1.46 ± 2.18 mm at target, and −1.76 ± 3.11 mm in depth. Potentially clinically relevant deviations (>2 mm entry/target or >10 mm depth) occurred in 6.6, 13.4, and 11.4% of electrodes, respectively. sEEG led to surgical therapy of various modalities in 27/53 patients (50.9%). Two patients required revision surgery due to significant deviations in electrode positioning (3.4% of surgeries) and one epidural hematoma needed surgical evacuation (1.7% of surgeries). Accuracy was strongly influenced by surgical workflow and surgeon experience, with a statistically significant improvement of depth accuracy (*p* = 0.0004). Progressive accuracy improvement over time indicated workflow refinement and procedural experience. The slotted cannula was not associated with a significant overall accuracy advantage.

**Conclusion:**

Frame-based sEEG implantation demonstrates high accuracy and safety. Deviations were predominantly minor and clinically acceptable, Accuracy appears primarily determined by surgical experience and workflow standardization rather than adjunctive devices. Selective cannula use may be reasonable in technically demanding trajectories, but routine application does not confer measurable benefit.

## Highlights

- Frame-based stereoelectroencephalography (sEEG) demonstrates high accuracy and safety.
- Surgical experience and workflow management were the strongest determinants of implantation accuracy, surpassing the effect of any single technical adjunct.
- Progressive procedural refinement is indicated by improvement in targeting precision and reduction of relevant errors over time.
- The slotted guiding cannula did not confer a general accuracy advantage but may support control in selected technically demanding trajectories.
- Ergonomic factors and procedural sequencing significantly influenced technical performance, emphasizing the need for structured workflow optimization in sEEG programs.

## Introduction

Stereoelectroencephalography (sEEG) is a critical diagnostic tool for patients with drug-resistant epilepsy (1–8). By enabling three-dimensional intracranial mapping of epileptogenic networks, sEEG supports tailored surgical and neuromodulatory treatment strategies (9). Precise electrode placement is critical: even minor deviations may compromise seizure localization or increase the risk of hemorrhagic or neurological complications.

Implantation accuracy is traditionally achieved using frame-based stereotactic systems or robotic assistance (10). While these platforms provide high geometric precision, technical variability may still arise from drilling, skull angle, depth control, and procedural sequencing. To enhance trajectory stability, adjunctive devices, such as a slotted guiding cannula, have been proposed to further enhance precision (11, 12). The cannula is intended to improve control during drilling and electrode advancement, particularly in tangential or technically demanding trajectories. However, its clinical benefit remains uncertain, and concerns have been raised regarding potential cortical or vascular tissue trauma (13). Beyond device-related factors, surgical experience and workflow organization may substantially influence implantation accuracy. Learning effects, procedural fatigue, and team coordination have received comparatively limited attention in literature, despite their potential impact on technical performance.

The present retrospective single-center study aims to evaluate the effectiveness and safety of the slotted guiding cannula during sEEG implantation In addition, we analyze workflow-related determinants, including surgical experience and implantation sequence, to assess whether implantation precision is primarily driven by adjunctive tools or by procedural standardization and operator expertise.

## Methods

After approval from the institutional review board of Ludwig Maximilians University of Munich (25-0543), the patient database of the Department of Neurosurgery was retrospectively searched for all consecutive patients who underwent stereoelectroencephalography (sEEG) implantation using the Leksell G-frame system (Elekta AB, Stockholm, Sweden) between September 2021 and May 2025. This single-center cohort included 59 operations performed in 53 patients, with a total of 678 electrodes implanted. Informed consent was obtained from all patients or their legal guardians. Prior to 2021, stereotactic procedures at our institution were primarily performed using a modified Riechert-Mundinger frame system (14). Owing to changes in regulatory requirements and improved hardware compatibility, the Leksell G-frame was subsequently introduced and fully adopted for all sEEG implantations. Importantly, only implantations performed with the Leksell G-frame were included in the present analysis. No procedures using the Riechert-Mundinger system were part of this study. All procedures were performed by three experienced functional neurosurgeons with stable team composition throughout the study period. The investigation therefore evaluates implantation accuracy exclusively within the new frame environment after its clinical introduction.

### Preoperative planning

**Figure 1:**
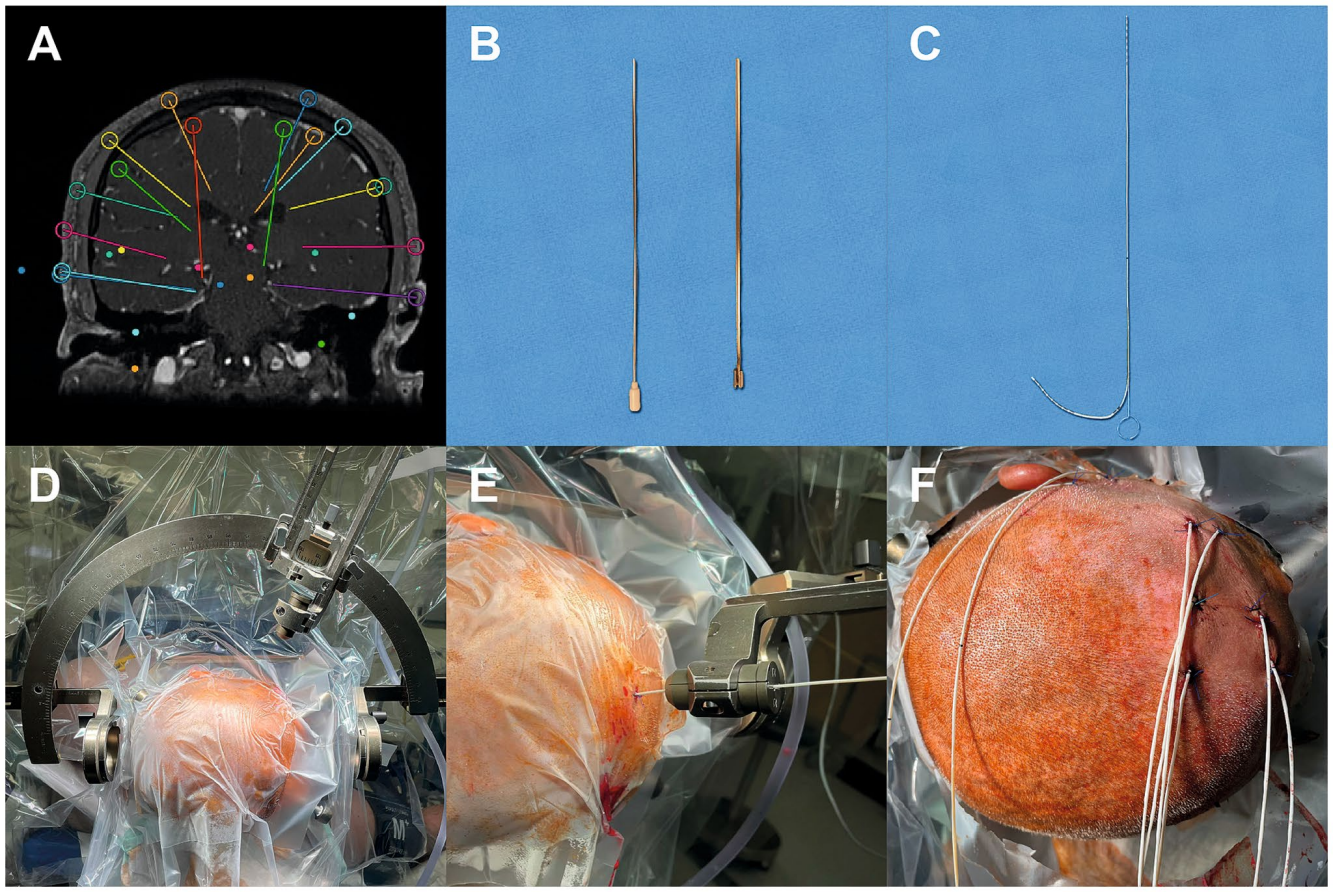
Surgical workflow for stereoelectroencephalography (sEEG) implantation. **(A)** Preoperative trajectory planning based on cranial magnetic resonance imaging. **(B)** Two-piece slotted cannula (Ad-Tech Medical Instrument Corporation, USA) with blunt-tipped stylet. **(C)** Multicontact depth electrode (Ad-Tech Medical Instrument Corporation, USA) with flexible stylet. **(D)** After disinfection and draping, the Leksell G frame (Elekta AB, Stockholm, Sweden) was mounted in a sagittal posterior position. **(E)** Electrode insertion along the planned trajectory. **(F)** Electrodes were secured at the skin level with a single stitch.

Former surface EEG data was used to decide which regions should be evaluated with further sEEG. Surgical planning was based on preoperative cranial magnetic resonance imaging (cMRI) scans (1.5- or 3.0-T scanners, Magnetom Symphony, Siemens, Erlangen, Signa HDxt, GE Healthcare, Little Chalfont, UK) including axial T2-weighted, axial FLAIR, and 3D T1-weighted sequences before and after intravenous gadolinium administration (0.1 mmol/kg) (Figure 1A) (15). When clinically indicated, positron emission tomography (PET) and single-photon emission computed tomography (16) were also incorporated. All imaging datasets were fused with a contrast-enhanced cranial computed tomography (cCT) angiography scan acquired with the stereotactic frame attached to the patient. This frame-based CT served as the geometric reference for final trajectory definition, thereby eliminating navigation registration error as a potential source of misalignment. Frame registration accuracy was routinely below 0.5 mm.

During the final planning step, all trajectories were verified directly within the frame-based CT dataset to confirm spatial consistency between coordinate calculation and imaging anatomy.

Trajectories were deliberately planned to avoid vascular structures, sulci, and cerebrospinal fluid spaces to minimize risks of hemorrhage and brain shift due to CSF loss (15, 17).

### Surgical procedure

All implantations were performed under general anesthesia using the Leksell G-frame (Elekta AB, Stockholm, Sweden). Patients’ hair was shaved and prophylactic antibiotic treatment was given, according to the institutional protocol. After frame fixation, an intraoperative contrast-enhanced cCT angiography scan was obtained and trajectory planning was finalized. After draping the frame coordinates for each electrode were set by the operating neurosurgeon and reviewed by a second neurosurgeon prior to incision. The first electrode was implanted following confirmation of all stereotactic settings, after which subsequent electrodes were sequentially placed using the same verification protocol. A 4–6 mm skin incision was made, followed by frame-guided burr hole trepanation (3 mm diameter). Burr holes were kept small, limiting CSF egress to only minimal droplets without clinically relevant intracranial volume change. The dura was perforated and electrode insertion was performed either directly or with the use of a slotted guiding cannula (Figures 1B,C), depending on trajectory and case-specific requirements (Figures 1D,E). The slotted guiding cannula was used intraoperatively when the surgeon perceived increased mechanical resistance during drilling or electrode advancement, or when minor deviation from the intended trajectory was suspected.

When the slotted guiding cannula was used, a 2.11-mm-diameter, two-piece slotted cannula (Ad-Tech Medical Instrument Corporation, USA) with a blunt-tipped stylet in place (Figure 1B) was slowly advanced through the cannula sleeve guide, into the brain, and along the planned electrode trajectory (12). Once the tip of the cannula had reached the estimated depth, the blunt stylet was removed while the cannula was held in place manually. A multicontact depth electrode (Ad-Tech Medical Instrument Corporation, USA) containing a thin flexible stylet (Figure 1C) was then inserted into the cannula until the electrode tip reached the end of the probe. Depth control was ensured by two independent intraoperative reference mechanisms. First, the intracranial electrode length up to the skin surface was calculated on the frame-based CT during planning and manually marked on the electrode shaft prior to insertion. Second, all electrodes carried a manufacturer-defined zero reference marking that was aligned intraoperatively with the calculated target depth marking on the stereotactic frame. This dual control system allowed continuous verification of insertion depth during advancement. Finally, the electrode stylet, slotted cannula, and frame guide tube were carefully removed, leaving the depth electrode in position.

Anchor bolts or bone screws (18) were not used for electrode fixation. Instead, electrodes were secured at the skin level with a single stitch (Figure 1F) (12) and a firm head wrap was applied postoperatively to reduce mechanical stress. The decision against bolt fixation was based on institutional practice and prior reports demonstrating stable monitoring without bolt systems (12, 14, 19). Given the presence of two independent intraoperative depth control mechanisms, additional intraoperative radiographic confirmation was not routinely performed in order to avoid unnecessary radiation exposure.

After electrode fixation and wound closure, the frame was removed and patients were monitored for immediate complications. Patients are sent to the epilepsy monitoring unit and subsequently monitored there. Prophylactic antibiotic therapy was administered during electrode implantation and continued for 3 days post-explantation.

### Postoperative imaging and accuracy assessment

**Figure 2:**
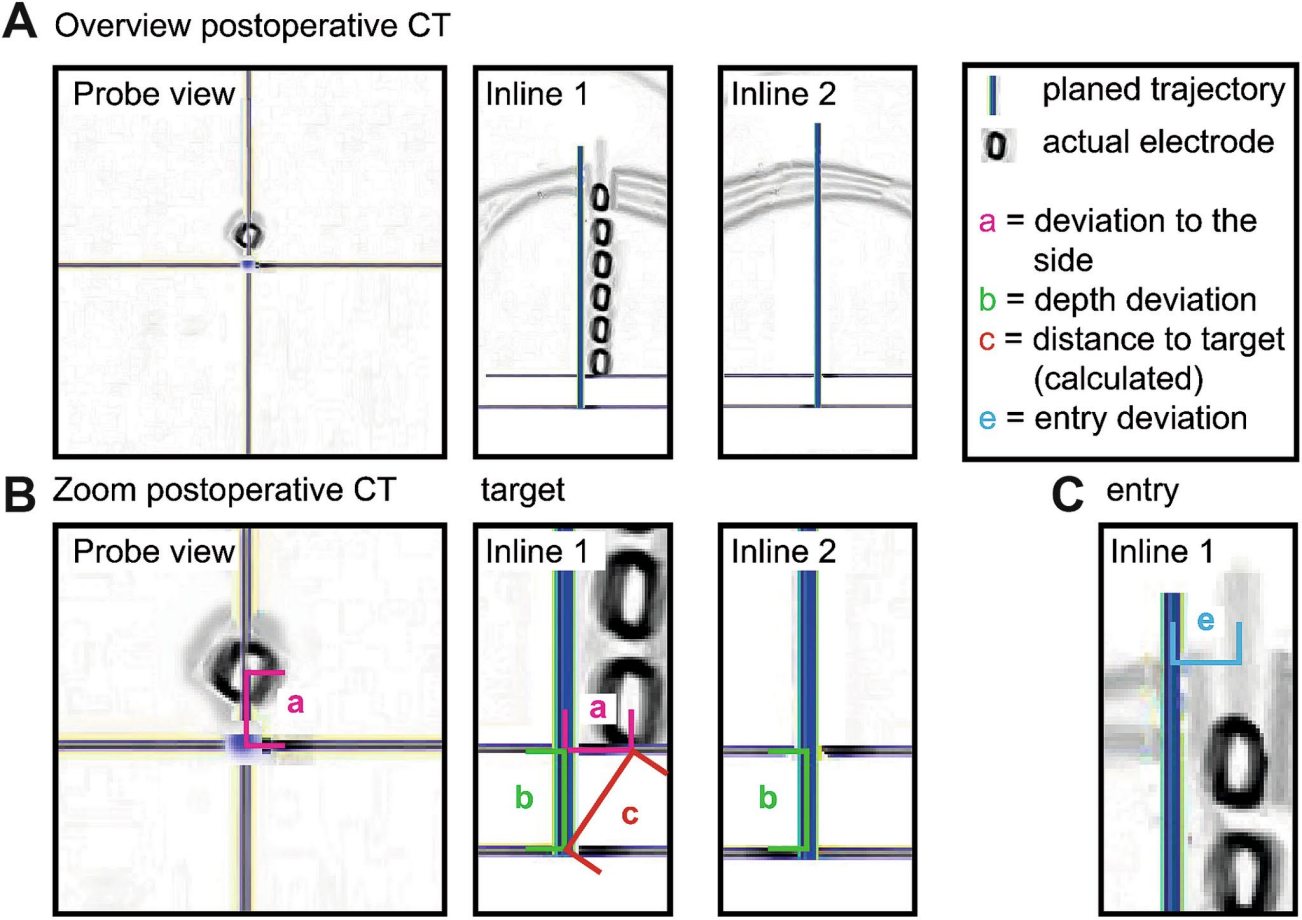
Measurement of electrode deviation on postoperative CT imaging. **(A)** The fused postoperative CT and preoperative planning dataset is rotated around the planned trajectory until the inline view aligns the imaging plane with the actual electrode axis. This orientation allows precise two-dimensional assessment along the trajectory. **(B)** Target region magnification: horizontal deviation from the planned target point **(a)** and depth deviation along the trajectory axis **(b)** are measured. The three-dimensional Euclidean distance to the target **(c)** is subsequently calculated from these components. **(C)** Entry region magnification: deviation at the entry point **(e)** is measured as the perpendicular distance between planned and actual entry coordinates.

**Figure 3:**
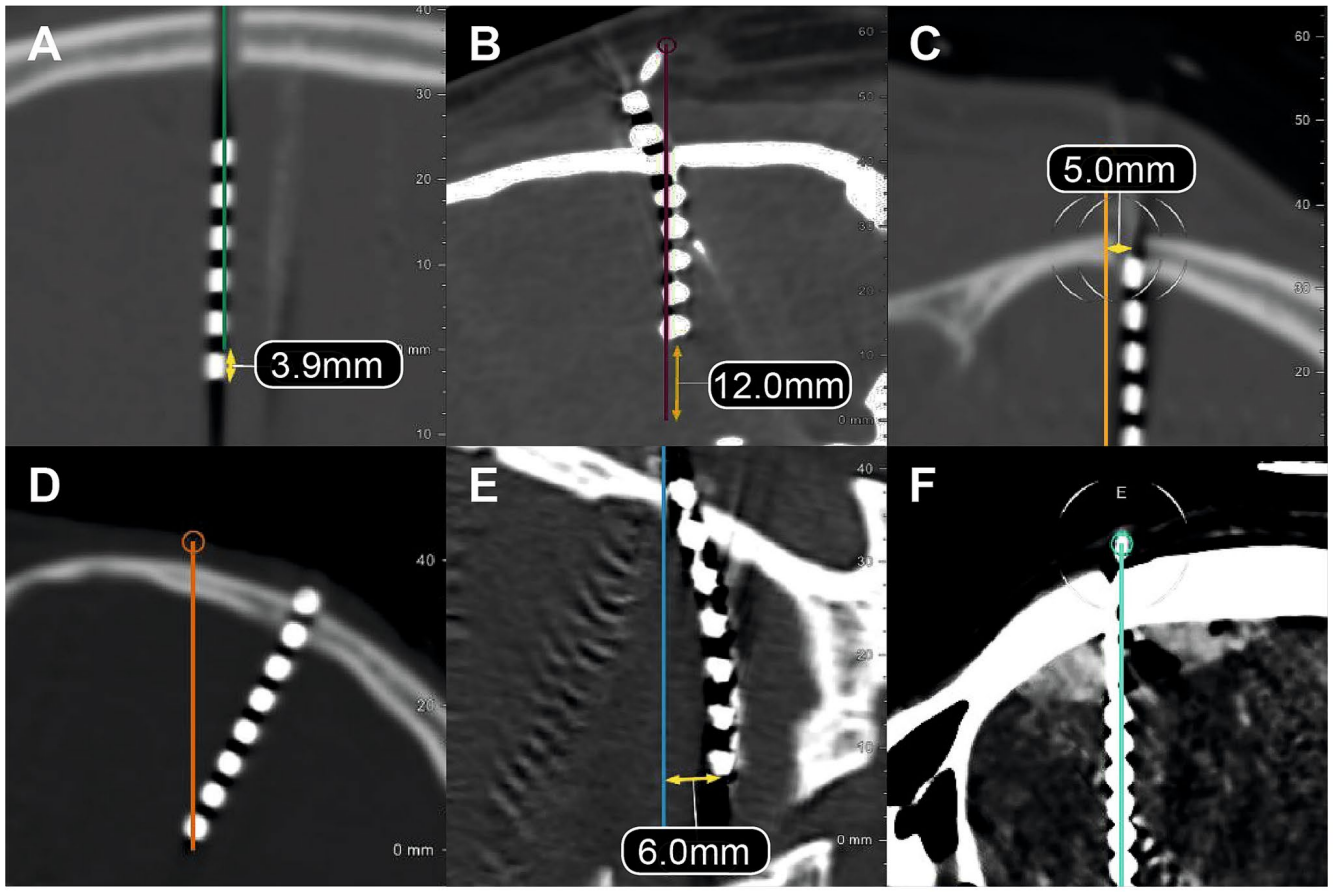
Different electrode placement error types. Postoperative CT scans fused with preoperative trajectory planning show various types of electrode placement errors. **(A)** Electrode placed too long, extending beyond the planned target. **(B)** Electrode placed too short, failing to reach the planned depth. **(C)** Entry point deviation resulting in an electrode positioned parallel to the planned trajectory due to an error in the X or Y coordinate. **(D)** Entry point deviation where the electrode still reaches the target due to a ring or arc angle error. **(E)** Deviation from the intended target due to trajectory misalignment. **(F)** Epidural hematoma observed postoperatively as a complication of implantation.

Postoperative cCT was routinely acquired within 24 h of implantation to exclude hemorrhage and fused with preoperative planning datasets to confirm electrode placement (Figure 2A). Depth deviation was determined relative to the planned electrode length (Figures 2B, 3A,B). Distances were measured at the entry point (Figures 2C, 3C) and target level between the tip of the electrode as visualized on postoperative imaging and the planned target point (Figures 2B, 3E) in the inline view. Euclidean distances between two points in a three-dimensional space were calculated using the Euclidean distance equation (20). Deviations from the planned trajectory were categorized as either harmless inaccuracies or potentially relevant errors. Potential relevance was defined as a deviation of more than 2 mm at the entry point or target, or a depth deviation of more than 10 mm. Because electrode contacts are distributed along a predefined segment rather than targeting a single focal point, minor depth deviations do not necessarily translate into loss of anatomical coverage. For this reason, only deviations exceeding 10 mm were classified as potentially clinically relevant. Since in these cases electrode contacts could miss the intended structure or harm could be done to surrounding tissue. Smaller deviations were considered harmless and did not require correction, even if visible on postoperative imaging.

Routine brain imaging was not performed immediately prior to electrode removal. However, no macroscopic electrode migration was observed clinically. At explantation, the depth markings of the electrodes consistently corresponded to the documented implantation depth at skin level, arguing against relevant postoperative displacement during the monitoring period. Furthermore, no systematic increase in deviation was observed in electrodes removed after longer monitoring intervals, making delayed migration an unlikely confounder of the postoperative accuracy assessment.

### Statistical analysis

The date of the first implantation served as the reference point, with the last follow-up recorded in September 2025. Continuous data are reported as mean (± standard deviation) and additionally as median with interquartile range where appropriate. Categorical variables are reported as frequencies (percentage). Group comparisons were performed using the chi-square test or Fisher’s exact test for categorical variables, and Student’s t-test for continuous variables. Given the non-normal distribution of deviation data, non-parametric testing using the Mann–Whitney *U* test was conducted. All statistical analyses were performed with SPSS Statistics [IBM, Version 29.0.0.0 (241)]. A *p*-value < 0.05 was considered statistically significant.

## Results

### Localization of electrodes

Electrode placement was distributed across several cortical regions. During 70% (41/59) surgeries electrodes were implanted in only one hemisphere, whereas in 30% (18/59) they were implanted in both hemispheres. The majority were located in the frontal lobe (37.9%), followed by the temporal lobe (31.7%) and parietal lobe (15.5%) (Table 1). Occipital (4.6%), insular (4.3%), and lesion-targeted (6.0%) implantations made up the remainder. This distribution reflects the diversity of epileptogenic networks investigated in presurgical evaluation (14, 21).

**Table 1:** Electrode deviation parameters by implantation site.

Localization	Deviation from entry (mm)	Horizontal distance to target (mm)	Distance to target (mm)	Deviation of depth (mm)
Frontal (*n* = 257)	0.33 ± 1.43	1.21 ± 2.12	2.71 ± 2.94	−1.44 ± 2.82
Insular (*n* = 29)	0.31 ± 1.04	2.00 ± 2.39	3.71 ± 2.72	−1.62 ± 3.02
Lesion (*n* = 41)	0.44 ± 1.12	1.88 ± 2.68	3.02 ± 3.29	−1.15 ± 2.83
Occipital (*n* = 31)	0.19 ± 0.60	1.13 ± 1.89	3.28 ± 2.86	−1.58 ± 3.44
Parietal (*n* = 105)	0.35 ± 0.87	1.69 ± 2.25	3.05 ± 2.90	−1.36 ± 2.84
Temporal (*n* = 215)	0.39 ± 1.14	1.54 ± 2.08	3.85 ± 3.17	−2.48 ± 3.47
Total (*n* = 678)	0.35 ± 1.20	1.46 ± 2.18	3.21 ± 3.05	−1.76 ± 3.11

Mean deviation values of all 678 implanted electrodes according to anatomical target regions. Data are presented as mean ± SD per electrode. Variability reflects trajectory length and target accessibility.

### Clinical follow-up

Of the 53 patients who underwent sEEG implantation, subsequent therapeutic procedures varied according to the diagnostic outcome. Twenty-two patients (41.5%) underwent resective epilepsy surgery, including one patient who received two resections after undergoing two sEEG investigations 3 years apart. Four patients (7.5%) received deep brain stimulation (DBS), and 1 patient (1.9%) was treated with a vagus nerve stimulator (VNS). Surgical intervention took place 11.2 ± 5.1 months after implantation. In 26 patients (49.1%), no surgical treatment option could be recommended based on the sEEG findings. The mean follow-up duration was 28.8 ± 11.5 months.

### Electrode placement accuracy and procedural characteristics

A total of 678 electrodes were analyzed. The number of electrodes per surgery ranged from 2 to 17. The minimum number of two electrodes occurred in a revision procedure, in which one previously misplaced electrode was removed and two new electrodes were implanted. The mean surgical duration was 103 ± 42 min.

Entry point deviation (Figures 3C,D) was present in 16.1% of electrodes, of which 6.6% exceeded the threshold for potential clinical relevance. At the target, 20.8% of electrodes showed a measurable deviation (Figures 3C,E), but only 13.4% were classified as potentially relevant errors. Depth inaccuracies (Figures 3A,B) were frequent but mostly within a harmless range: 34.2% of electrodes were slightly shorter than planned, while 4.3% exceeded the intended depth.

The pattern of deviations suggests that mechanical factors at the skull-dura interface played a major role. Entry point deviations were more commonly observed in electrodes with tangential trajectories, consistent with drill skidding on the calvarial surface or minor dural deflection during penetration (Figure 3E). Depth deviations were predominantly negative (electrodes too short), supporting the assumption of cautious advancement rather than uncontrolled overshooting. No systematic pattern indicative of postoperative migration was observed.

The majority of electrodes, 83.9% at the entry point, 79.2% at the target, and 61.5% for depth, were placed without measurable deviation (Table 2). Overall, 93.4% at the entry point, 86.6% at the target, and 88.6% for depth, were placed without clinically potentially meaningful error.

**Table 2:** Accuracy of electrode placement: distribution of potentially relevant errors.

Parameter	Category	Inaccuracy (*n*, %)	Potentially relevant error (*n*, %)	Total (*n*, %)
Entry deviation	Yes	64 (9.4%)	45 (6.6%)	109 (16.1%)
	No			569 (83.9%)
Deviation from target	Yes	50 (7.4%)	91 (13.4%)	141 (20.8%)
	No			537 (79.2%)
Depth deviation	Yes, too short	165 (24.3%)	67 (9.9%)	232 (34.2%)
	Yes, too long	19 (2.8%)	10 (1.5%)	29 (4.3%)
	No			417 (61.5%)

Summary of deviations at dura entry, target, and depth levels. Potentially relevant errors were defined as >2 mm deviation at entry or target, or >10 mm difference in insertion depth (too short or too long).

### Quantitative accuracy metrics

Detailed analysis of placement accuracy showed that the mean deviation from the entry point was 0.35 ± 1.20 mm, with a maximum of 20 mm. The mean horizontal distance to the target was 1.46 ± 2.18 mm, with a maximum deviation of 17 mm. The overall mean Euclidean distance to the target was 3.21 ± 3.05 mm, ranging up to 20 mm. The mean deviation of depth was −1.76 ± 3.11 mm, with a range between −20 mm (too short) and +12 mm (too long) (Table 3). These metrics demonstrate high placement accuracy with occasional outliers, underscoring the importance of systematic error prevention strategies.

**Table 3:** Electrode deviation parameters with and without slotted cannula use.

Cannula use	*N*	Deviation from entry (mm)	Horizontal distance to target (mm)	Distance to target (mm)	Deviation of depth (mm)
No	606	0.37 ± 1.24	1.48 ± 2.15	3.25 ± 3.05	−1.80 ± 3.13
Yes	72	0.17 ± 0.69	1.33 ± 2.41	2.87 ± 3.18	−1.42 ± 2.98
*p*-value (Mann Whitney)		0.061	0.128	0.203	0.473
Total	678	0.35 ± 1.20	1.46 ± 2.18	3.21 ± 3.05	−1.76 ± 3.11

Comparison of mean electrode deviation values depending on whether a slotted guiding cannula was used. Values are presented as mean ± SD. No statistically significant differences were observed. Statistical comparisons were performed using Mann–Whitney.

Subgroup analyses revealed no statistically significant differences in deviation parameters for electrodes with an intracranial length ≥50 mm (10% of electrodes) compared to shorter trajectories, nor for extreme skull thickness categories (>10 mm, 14%; <4 mm, 6%). However, a trend toward improved accuracy was observed in longer electrodes (horizontal deviation 0.86 ± 1.93 mm; depth deviation −1.10 ± 2.81 mm) and in cases with thicker bone (horizontal deviation 0.75 ± 1.60 mm; depth deviation −0.89 ± 2.35 mm), whereas electrodes implanted through thinner bone showed comparatively larger deviations (horizontal 2.25 ± 1.98 mm; depth −3.00 ± 2.63 mm). The mean intracranial electrode length was 37.8 mm (range 10 to 76 mm), and skull thickness ranged from 2 to 15 mm (mean 7.2 mm). These observations did not reach statistical significance and should therefore be interpreted cautiously.

### Complications and corrective measures

Complications were observed in a minority of cases. Two patients (3.4% of surgeries, 0.3% of electrodes) developed epidural hematoma, one of which required surgical evacuation. One patient (1.7% of surgeries, 0.15% of electrodes) suffered an intracerebral hemorrhage that was managed conservatively.

Regarding electrode placement, two patients required revision surgery due to significant deviations in electrode positioning, corresponding to 3.4% of surgeries (0.3% of electrodes). The two revision surgeries performed in this cohort were primarily due to significant spatial misalignment rather than isolated minor depth discrepancies. In addition, one case (1.7% of surgeries) required additional coverage after the initial implantation, leading to the insertion of three further electrodes 11 days later. In total, 53 patients underwent 59 stereotactic implantations. One implantation was intentionally split into two sessions due to widely separated target regions and technical considerations. Two patients underwent repeat implantations at later time points, one three years and the other one year after the initial procedure, due to the identification of new epileptogenic foci. Importantly, no complications related to infection or cerebrospinal fluid (CSF) leakage were observed in this cohort.

Taken together, the rate of clinically relevant complications directly attributable to electrode implantation was low, affecting less than 5% of surgeries, and most events were manageable without long-term sequelae. Regarding the total number of implanted electrodes (*n* = 678), the risk of a major complication per electrode was 0.3%, assuming that a single electrode is responsible for each hemorrhagic complication.

These results suggest a low but non-negligible risk of requiring additional intervention following the initial procedure, which is consistent with previously reported complication rates in sEEG (22, 23).

### Slotted cannula and technical modifiers

Analysis of cases using the slotted guiding cannula showed no statistically significant improvement in accuracy metrics (Table 3). The frequency of potentially relevant errors remained unchanged. While mean depth deviation tended to decrease from −1.81 mm to −1.42 mm (*p* = 0.63), the proportion of electrodes implanted too deep showed an increase from 4.1 to 5.6% (*p* = 0.28), both nonsignificant.

**Figure 4:**
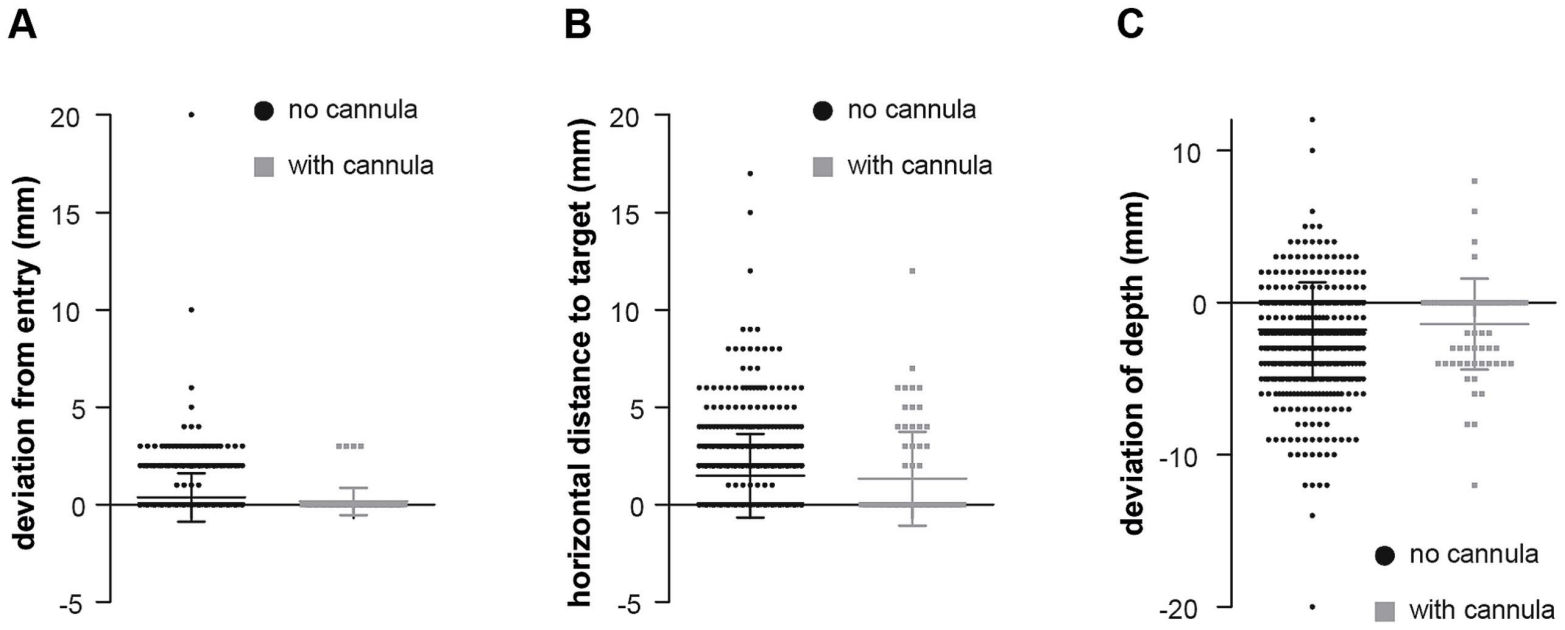
Effect of slotted guiding cannula on electrode placement accuracy. Comparison of electrodes implanted with and without the use of the slotted guiding cannula. **(A)** Mean deviation from the planned entry, **(B)** target, showing no significant superiority of the device. **(C)** Error in depth placement, indicating a potential increased risk of over-insertion when using the cannula. Mean + SD.

Of 215 temporal lobe electrodes, the slotted cannula was used in 28 cases (13.0%). Of 434 electrodes implanted in other regions, the cannula was used in 44 cases (10.1%). Table 1 presents deviation parameters stratified by implantation site. Given the limited number of cannula cases and the non-randomized intraoperative decision-making process, the dataset was not sufficiently powered for multivariable regression analysis to adjust for anatomical region, trajectory characteristics, or implantation order. Trends suggested that the cannula may be particularly helpful in cases requiring tangential entry angles, where it appeared to reduce deviations at dura penetration. At the same time, its use was associated with a tendency toward increased depth overshoot, suggesting a balance between improved trajectory alignment and the risk of over-insertion (Figure 4). These observations are consistent with previous reports suggesting a selective rather than a universal benefit of guiding cannulas for technically demanding trajectories.

### Learning curve and experience effects

**Figure 5:**
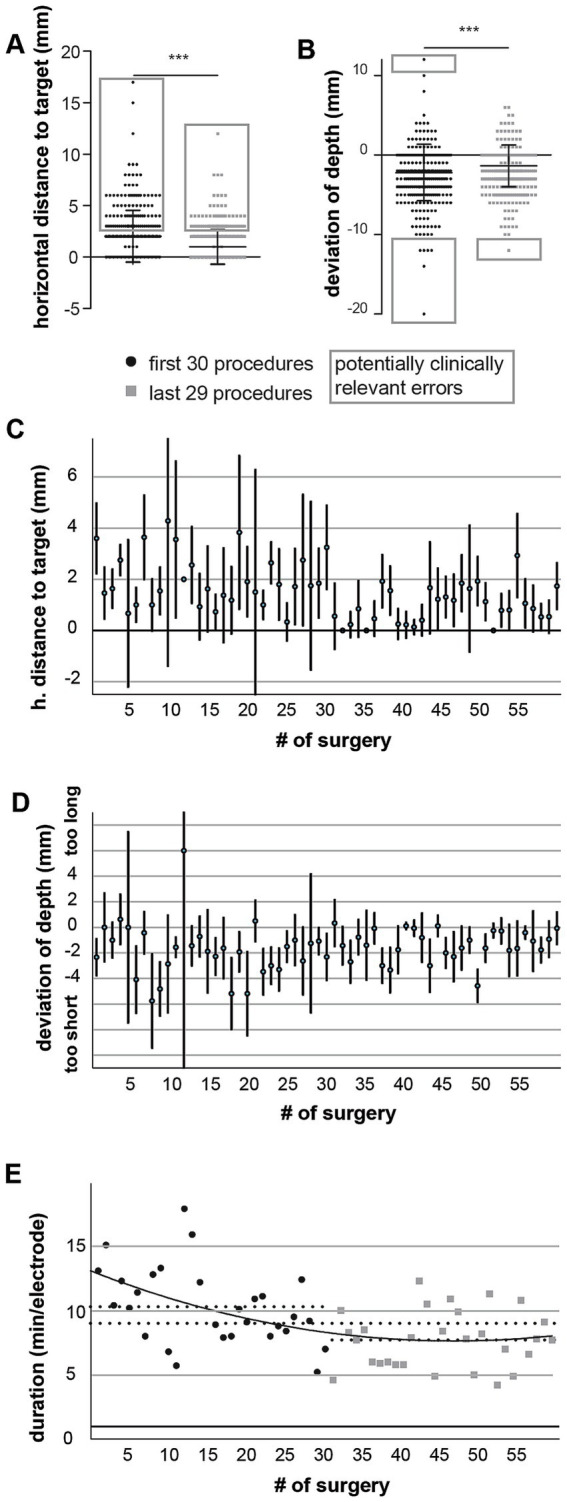
Learning curve: Improvement in implantation accuracy and procedural efficiency over time. **(A)** Mean horizontal distance to the target and **(B)** mean depth deviation are plotted for electrodes implanted in each surgery, demonstrating a downward trend over time toward improved accuracy as surgical experience increased. Error bars depict 95% confidence intervals (CI). **(C,D)** Comparison between the first 30 and last 29 surgeries shows significant improvement in both horizontal distance to the target (2.03 ± 2.52 mm vs. 0.98 ± 1.69 mm, *p* < 0.0001) and depth deviation (−2.21 ± 3.55 mm vs. −1.37 ± 2.62 mm; *p* = 0.0004), while deviation from entry remained unchanged (0.34 ± 1.48 mm vs. 0.36 ± 0.90 mm, n.s., not shown). The overall rate of potentially relevant errors decreased from 32 to 20% (*p* = 0.0004). **(E)** Mean implantation time per electrode across the study period demonstrates progressive procedural efficiency. The overall mean implantation time was 9.11 ± 2.93 min per electrode (range 4.3–18 min). Implantation time decreased significantly between the first 30 and last 29 surgeries (10.42 ± 3.00 min vs. 7.81 ± 2.23 min per electrode, *p* = 0.0004).

Comparison of the first 30 with the subsequent 29 surgeries demonstrated a significant improvement in targeting accuracy over time (Figures 5A,B). In the early group, mean depth deviation was −2.21 mm (SD 3.55) compared with −1.37 mm (SD 2.62) in later cases (*p* = 0.0004). Similarly, the mean horizontal distance to target improved from 2.03 mm (SD 2.52) to 0.98 mm (SD 1.69; *p* < 0.0001). Deviation from entry remained stable (0.34 mm vs. 0.36 mm, n.s.). The proportion of potentially relevant errors also decreased from 32% in the early cohort to 20% in the later cohort (*p* < 0.01).

A clear learning curve was observed, with progressive improvement in electrode placement accuracy and procedural efficiency over the course of the study period. Surgeries performed later in the timeline showed reduced mean deviations from both entry point and final target, supporting the effect of accumulated experience and refined workflow practices (Figures 5C,D). The mean implantation time per electrode across all surgeries was 9.11 ± 2.93 min (range 4.3 to 18 min). Implantation time decreased significantly from 10.42 ± 3.00 min to 7.81 ± 2.23 min per electrode (*p* = 0.0004), when comparing the first 30 surgeries with the subsequent 29 (Figure 5E). These findings indicate a progressive refinement of technique and workflow translating into greater accuracy and fewer clinically relevant deviations as surgical experience accumulated. While accuracy metrics continued to improve gradually throughout the study period without a clear plateau, implantation speed showed a more distinct stabilization after approximately 30 procedures, suggesting the completion of an initial technical learning phase. All procedures during the study interval were performed by a stable surgical team. The study period concluded due to subsequent personnel changes, and therefore further case accumulation beyond this point was not included in the present analysis.

### Implantation sequence and error distribution

**Figure 6:**
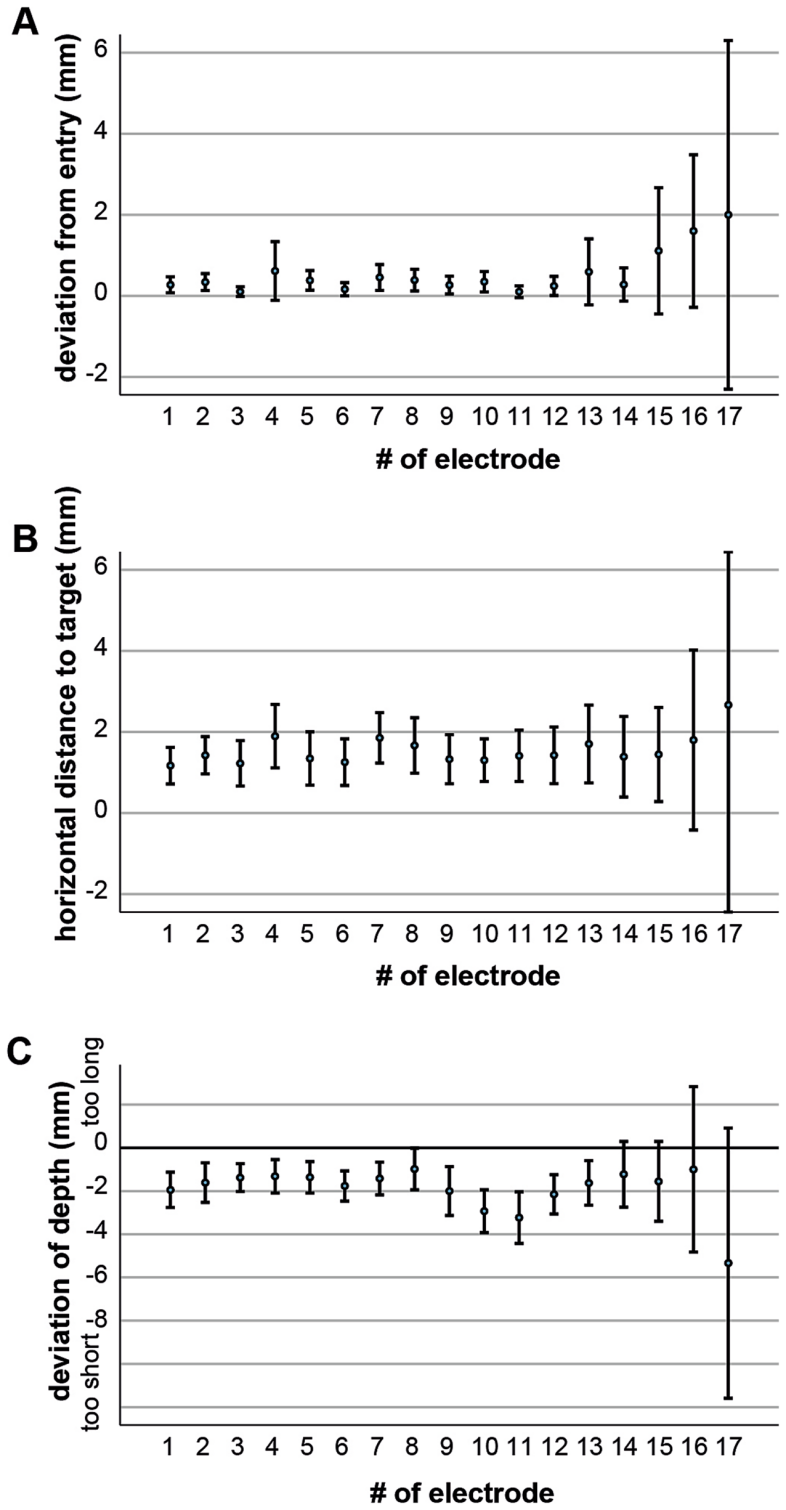
Implantation sequence effects electrode accuracy. Mean deviation from entry point **(A)**, horizontal distance to target **(B)**, and depth deviation **(C)** are displayed according to electrode implantation order within each surgery. Later-placed electrodes showed slightly higher deviations, suggesting a potential influence of surgeon fatigue and reduced concentration over time. Error bars depict 95% CI.

An analysis of implantation order demonstrated higher deviation rates in electrodes placed later during the procedure (Figure 6). This pattern is compatible with a time-dependent decline in sustained concentration or increased cognitive load over the course of prolonged stereotactic workflows. In our standard operative sequence, temporal electrodes were typically implanted last. This order was chosen for ergonomic reasons, as earlier placement would have obstructed access to other trajectories once the head was rotated 90° for lateral approaches. Only few exceptions to this sequence occurred, and their number was insufficient for meaningful subgroup analysis.

The increased deviation observed in temporal electrodes is therefore potentially multifactorial. Entry point deviations are most plausibly explained by procedural sequence effects, including reduced concentration or accumulated procedural strain toward the end of surgery. In addition, intraoperative mechanical resistance from the temporalis muscle, particularly in tangential trajectories, may contribute to minor drill skidding or limited visualization at the skull surface.

Depth deviations, in contrast, may be influenced by postoperative soft-tissue swelling and muscle tension, especially in the temporal region where electrodes traverse thicker musculature and are subject to wound closure dynamics.

Given that temporal electrodes were predominantly implanted at the end of the sequence, a complete statistical separation of sequence-related and region-specific effects is not feasible in this retrospective design. The observed pattern likely reflects an interaction between procedural order and regional anatomical factors rather than a single dominant mechanism.

## Discussion

The transition to the Leksell G frame system in 2021 marked a change in stereotactic hardware and workflow at our institution. Although the surgical team had extensive prior experience with frame-based sEEG, the introduction of a new coordinate system, mechanical setup, and instrumentation required procedural adaptation. The present study therefore does not describe a learning curve for sEEG as a surgical concept, but rather the adaptation of an experienced epilepsy surgery team to a modified stereotactic environment.

Frame-based sEEG is fundamentally a mechanically defined procedure in which trajectory orientation is determined by calculated coordinates and frame geometry. In this context, entry point accuracy largely depends on correct coordinate transfer and stable frame setup, with limited influence of manual dexterity. The absence of improvement in entry deviation over time in our series is consistent with this principle and supports the mechanical reliability of the coordinate system once properly applied.

The surgical team sought to refine implantation techniques and explored adjunctive tools such as the slotted guiding cannula to enhance trajectory control and compensate for perceived workflow-related limitations. Cannula use was triggered intraoperatively when increased mechanical resistance during drilling or electrode advancement was perceived, rather than based on predefined trajectory criteria. While tangential trajectories appeared more likely to prompt cannula use, the distribution across anatomical regions was comparable. Due to the limited number of cannula-assisted implantations and the retrospective design, adjustment for potential confounders such as trajectory angle, length, or skull thickness was not statistically robust. Therefore, the observed associations should be interpreted cautiously.

While the device was designed to improve control at the dura interface it also introduced a trend toward excessive depth insertion. This dual effect highlights the importance of selective rather than routine use, applying the cannula only where trajectory angles justify the added manipulation.

Although anchor bolt fixation is widely used in many centers (18), stable electrode positioning can be achieved without bolt systems when meticulous intraoperative depth control and postoperative immobilization are ensured (12, 14, 24). In our cohort, two independent depth reference mechanisms were applied during implantation, and no clinical evidence of postoperative migration was observed. The predominance of small negative depth deviations suggests cautious underinsertion rather than secondary displacement. Moreover, the absence of progressive deviation patterns argues against systematic postoperative migration as a relevant confounder of the reported accuracy metrics.

It should also be noted that in sEEG, electrode contacts are distributed along a predefined trajectory segment, and millimetric depth variation does not necessarily compromise diagnostic yield. Depth control therefore does not reflect a binary target-hit paradigm. Importantly, the concept of accuracy in sEEG implantation should be interpreted within the clinical objective of electrode placement. Unlike functional stereotactic procedures targeting a single anatomical point, sEEG electrodes are typically intended to sample predefined anatomical regions based on three-dimensional hypotheses of epileptogenic networks. Consequently, implantation follows established anatomical trajectory patterns rather than aiming for millimeter-level precision at a single coordinate (25). From a clinical perspective, the primary goal of high stereotactic accuracy is therefore to ensure safe trajectory execution and reliable sampling of the intended brain regions while minimizing the risk of vascular or cortical injury (20).

Entry accuracy and overall performance were found to be strongly influenced by the human factor. Surgical precision in sEEG depends not only on the surgeon’s technical skill but also on sustained concentration and coordinated teamwork. Our data indicate a progressive decline in accuracy over the course of a procedure, suggesting that fatigue and reduced vigilance contribute to cumulative minor deviations. This finding underscores that even with precise preoperative planning, human limitations remain a key source of intraoperative variability. Adopting structured breaks, optimizing electrode order, and maintaining consistent intra-team communication (e.g., the “four-eye” verification principle) may mitigate this drift in concentration. These approaches align with ergonomic studies in neurosurgery emphasizing cognitive load management and workflow optimization (24, 26, 27).

Training and experience also play central roles in achieving consistent accuracy (28). Prior reports recommend that sEEG programs be established in centers with at least 5 years of dedicated experience (6), ensuring adequate exposure to a range of anatomical challenges and trajectory planning scenarios. Our results support this recommendation and illustrate a learning phase following the introduction of a new stereotactic system in an already experienced center. Accuracy improved progressively over time without reaching a clear plateau, while procedural efficiency stabilized after approximately 30 surgeries, suggesting early workflow consolidation followed by continued refinement of targeting precision. Data collection was intentionally limited to the period before personnel changes occurred to maintain methodological consistency. Consequently, the learning curve presented here reflects performance evolution within a stable surgical team rather than long-term institutional outcomes.

Compared to published data, our accuracy metrics are consistent with those reported in previous studies (18, 20, 29, 30), though we observed slightly greater variability in depth deviation. This may be explained by differences in fixation techniques, as our workflow does not employ bolts or intraoperative radiographic verification. While frameless systems and robotic assistance have demonstrated potential for improving orientation and reproducibility (31), their reported accuracy varies depending on platform design and registration technique. Recent clinical experience with table-mounted robotic alignment systems such as the Cirq robotic arm has shown promising implantation precision in sEEG, supporting the feasibility of compact robotic assistance within conventional operating room workflows (19, 31, 32). Similarly, robot-assisted systems such as ROSA have demonstrated high targeting accuracy, although deviations are influenced by the chosen referencing method, with frame-based CT registration generally providing superior precision compared to facial laser scan-based techniques (33). Moreover, recent analyses of frameless sEEG implantation using stepwise workflow refinement and appropriate statistical modelling of nested electrode data have highlighted that accuracy improvements are not solely technology-driven but also process-dependent (34).

In contrast to robot-assisted or navigation-based systems, frame-based stereotaxy eliminates navigation registration mismatch as a relevant error source once the frame CT is acquired. In our workflow, all final trajectory planning was performed within the frame-referenced CT dataset, and registration accuracy was consistently below 0.5 mm. Consequently, deviation mechanisms related to optical tracking or surface-based registration, as described in robotic series, are not applicable to the present cohort (19, 31–34).

Thus, while robotic and frameless approaches may enhance reproducibility and streamline trajectory alignment, their benefit remains contingent on optimal hardware-software integration and structured workflow implementation. As navigation and robotic platforms continue to evolve, tighter coupling with planning software and standardized procedural protocols may further enhance safety and efficiency by reducing manual setup steps and alignment errors (10, 19, 23, 31, 32, 34, 35).

Intraoperative imaging-based depth verification has been proposed as an adjunct safeguard in robotic or frameless workflows (31). In a purely frame-based system, however, trajectory orientation and insertion depth are mechanically defined by coordinate geometry once the frame is fixed and the guide is locked. In our workflow, depth control relied on two independent intraoperative reference mechanisms, providing redundant mechanical verification during electrode advancement.

The predominance of small negative depth deviations in our cohort suggests cautious underinsertion rather than systematic inaccuracy or uncontrolled overshooting. Clinically meaningful depth errors were rare. Notably, the two revision surgeries observed in this series resulted from incorrect coordinate setting rather than isolated depth miscalculation; therefore, intraoperative fluoroscopy would not have prevented these cases. Although fluoroscopy may assist with trajectory visualization, it provides only limited information on three-dimensional targeting accuracy and therefore cannot be considered equivalent to volumetric imaging for precise deviation assessment. In contrast, intraoperative CT might have detected spatial misalignment prior to wound closure (31). In many centers, intraoperative CT requires patient transport while intubated or access to specialized imaging infrastructure that is not universally available. These additional logistical steps increase resource utilization and may introduce potential risks related to patient transport, anesthesia duration, and workflow disruption. We deliberately decided against routine intraoperative CT imaging also because of radiation exposure (14), particularly given the relatively young patient population in this cohort (mean age 29 years). In the context of the high implantation accuracy and low revision rate observed in this series, routine intraoperative CT verification may therefore not be necessary in all cases, particularly in experienced centers using standardized stereotactic workflows. Selective use of intraoperative CT in complex cases may represent a reasonable compromise, especially in centers that do not employ bolt fixation. Overall, our findings suggest that minimizing implantation error in sEEG requires a multifactorial approach: targeted use of technical adjuncts, structured team communication, fatigue management, and sustained training within experienced centers. Future prospective studies should evaluate how standardized protocols for rest, workload distribution, and team coordination influence long-term accuracy, safety, and seizure outcomes (8, 36).

## Conclusion

Accurate electrode placement in frame-based stereoelectroencephalography depends primarily on surgical experience, workflow optimization, and cognitive endurance rather than on the routine use of additional guiding devices. While the slotted guiding cannula may offer selective advantages in technically demanding trajectories, its overall contribution to accuracy was limited. The observed learning curve underscores the importance of accumulated experience, structured team coordination, and fatigue management to maintain precision throughout the procedure. A multifactorial approach integrating technical expertise, ergonomic workflow design, and selective tool use is essential to ensure both accuracy and safety in sEEG implantation. Future prospective studies should further define standardized strategies for workflow optimization and team training to enhance reproducibility and minimize risk across centers.

### Patient consent

Informed consent for all surgical procedures was obtained from all patients or their legal guardians. Where applicable, explicit consent was obtained for the use of anonymized data and imaging in this study.

## Data Availability

The raw data supporting the conclusions of this article will be made available by the authors, without undue reservation.
